# Minimally Invasive Repair of Left Ventricular Aneurysm After Esophagectomy With Retrosternal Gastric Conduit: A Case Report

**DOI:** 10.1002/ccr3.71548

**Published:** 2025-11-27

**Authors:** Junya Kitaura, Tomokuni Furukawa, Kazuki Maeda

**Affiliations:** ^1^ Department of Cardiovascular Surgery Akane‐Foundation Tsuchiya General Hospital Hiroshima Japan

**Keywords:** cardiopulmonary bypass, esophagectomy, heart aneurysm, minimally invasive surgical procedure, thoracotomy

## Abstract

In patients with prior esophagectomy and retrosternal gastric conduit, conventional sternotomy may be hazardous. Left mini‐thoracotomy provides a safe and effective alternative for repairing left ventricular aneurysm, highlighting the importance of tailoring the surgical approach to individual anatomical challenges.

## Graphical abstract

Preoperative CT and 3D reconstruction demonstrating an inferior left ventricular aneurysm in a patient with a retrosternal gastric conduit, highlighting the rationale for choosing a left mini‐thoracotomy approach.

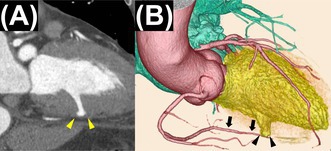

## 1 Introduction

Left ventricular aneurysm (LVA) is an uncommon but clinically important condition, most often occurring after myocardial infarction but also associated with other cardiac and systemic diseases [1, 2]. Standard surgical repair is usually performed through a full median sternotomy [1]. Herein, we describe a successful minimally invasive cardiac surgery (MICS) via left mini‐thoracotomy in a patient with prior esophagectomy and retrosternal gastric conduit reconstruction, where sternotomy was undesirable.

## 2 Case History

An 80‐year‐old man with a history of esophagectomy and retrosternal gastric conduit reconstruction for esophageal cancer 28 years earlier experienced exertional dyspnea and chest pain while abroad. Although his symptoms improved with conservative management, he visited our hospital for further evaluation after returning home.

At presentation, his vital signs were as follows: body temperature 36.8°C, blood pressure 154/92 mmHg, heart rate 80 beats/min, and oxygen saturation 97% on room air. No jugular venous distension, lower‐extremity edema, or cardiac murmur was observed. A midline abdominal surgical scar was noted. Laboratory tests revealed an N‐terminal pro–B‐type natriuretic peptide (NT‐proBNP) level of 2765 pg/mL and a high‐sensitivity troponin I concentration of 42.9 pg/mL. There was no history of thoracic trauma or recent chest injury.

**FIGURE 1:**
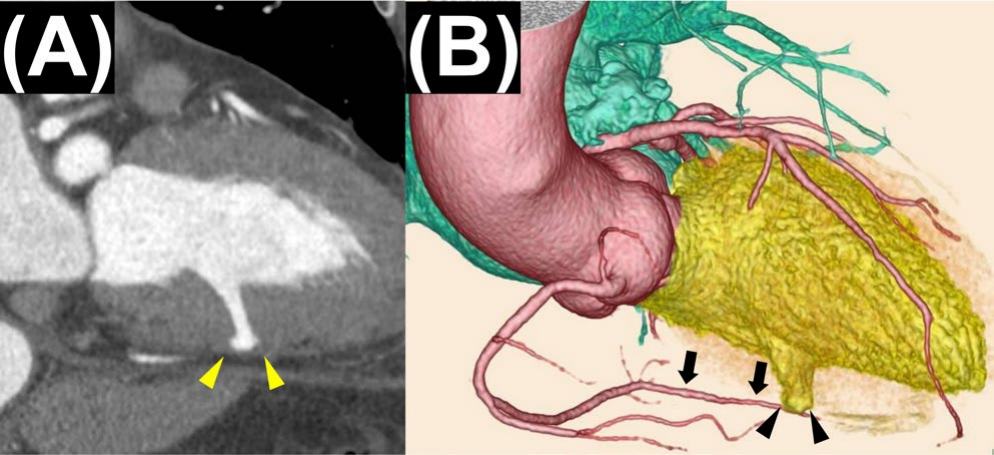
Preoperative CT images. (A) Long‐axis view demonstrating an LVA (arrowheads). (B) Three‐dimensional reconstruction showing the LVA (arrowheads) and the course of the right coronary artery to its distal portion (arrows). CT, computed tomography; LVA, left ventricular aneurysm.

Coronary computed tomography (CT) angiography revealed a saccular aneurysm in the basal‐to‐mid inferior wall of the left ventricle (LV), with an approximately 8‐mm neck and discontinuity of the myocardial layer (Figure 1A,B). However, there was no evidence of obstructive coronary artery disease, ischemic changes, or prior myocardial infarction. Cardiac magnetic resonance imaging demonstrated preserved myocardial signal intensity without delayed gadolinium enhancement. Preoperative transthoracic echocardiography (TTE) showed no regional wall motion abnormality, indicating relatively preserved global LV contractility. Transesophageal echocardiography (TEE) was not performed because of the prior esophagectomy with retrosternal gastric conduit reconstruction.

Two months earlier, no pericardial effusion had been detected on plain chest–abdominal CT; however, new‐onset pericardial effusion was observed at presentation. Given the concern for impending rupture of the lesion, surgical intervention was deemed necessary.

Median sternotomy was considered highly challenging due to the presence of a retrosternal gastric conduit, which posed a substantial risk of conduit injury, dense adhesions, bleeding, and infection. Therefore, we selected an MICS approach via left mini‐thoracotomy. The LVA had a fissure‐like morphology, and direct suture closure was planned with particular care to preserve the right coronary artery.

## 3 Treatment

### 3.1 Surgical Procedure

**FIGURE 2:**
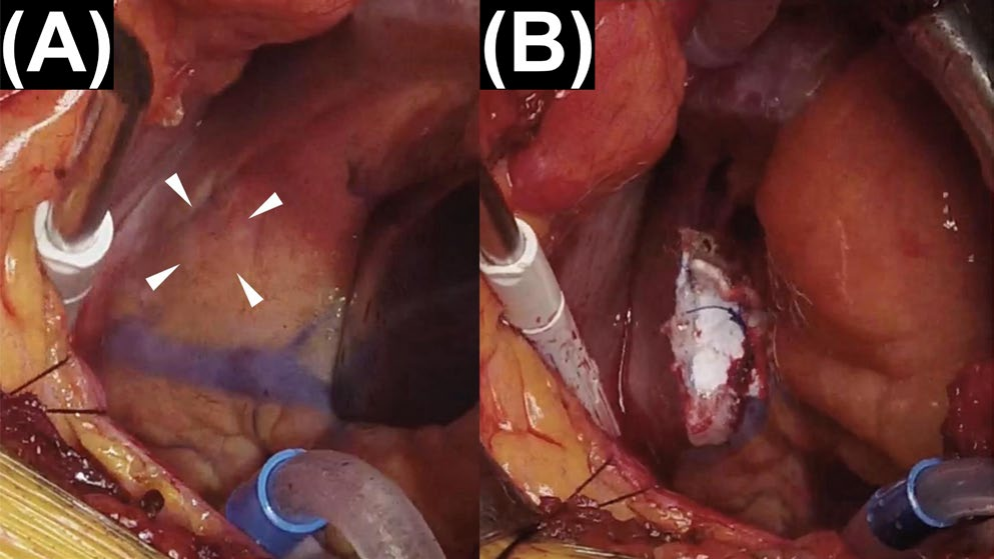
Intraoperative images. (A) LVA in the inferior wall (arrowheads). (B) Aneurysm closed with direct sutures, buttressed with 10 × 20 mm felt pledgets and reinforced with Hydrofit.

Under general anesthesia with a double‐lumen endotracheal tube to permit lung isolation, the patient was positioned in the right semi‐lateral decubitus position with the left arm lowered alongside the body. A left mini‐thoracotomy was performed in the sixth intercostal space to expose the inferior LV wall. No obvious bulging was observed on visual inspection (Figure 2A). After ventricular filling, a dyskinetic area was identified in the distal right coronary artery territory. Epicardial echocardiography clearly identified a localized LVA at the inferior wall. The operative field revealed no adhesions or pericardial scarring, consistent with a nonischemic etiology. Cardiopulmonary bypass (CPB) was established via left femoral arterial cannulation and right femoral venous drainage. The inferior LVA was closed using 3–0 polypropylene mattress sutures buttressed with 10 × 20 mm felt pledgets (Figure 2B). Particular care was taken to preserve the right coronary artery. The closure site was additionally reinforced externally with Hydrofit (Sanyo Chemical Industry, Kyoto, Japan). The total operative time was 120 min, and the CPB time was 48 min. Because the operation was performed on a beating heart, histopathologic confirmation was not feasible.

## 4 Results (Outcome and Follow‐Up)

**FIGURE 3:**
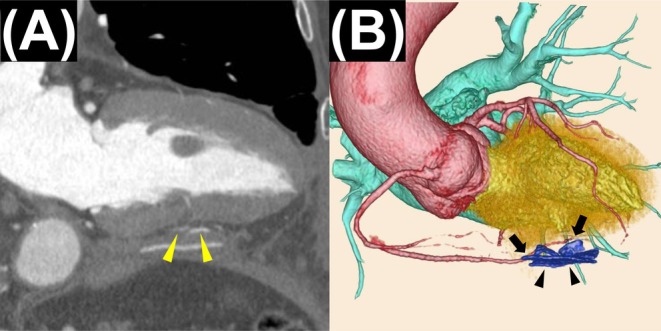
Postoperative CT images. (A) Long‐axis view demonstrating complete exclusion of the aneurysm cavity (arrowheads). (B) Three‐dimensional reconstruction showing complete exclusion of the aneurysm cavity (arrowheads) and preserved course of the right coronary artery to its distal portion (arrows).

The postoperative course was uneventful, and the patient was discharged on postoperative day 17. Postoperative CT confirmed complete exclusion of contrast filling into the inferior LVA cavity (Figure 3A,B). At the 6‐month follow‐up, no recurrence was observed.

## 5 Discussion

Although LVA most commonly occurs after myocardial infarction [1], other acquired causes have also been reported, including arrhythmogenic right ventricular cardiomyopathy, hypertrophic cardiomyopathy, myocarditis, and trauma, as well as noncardiac or systemic diseases such as sarcoidosis, Chagas disease, systemic lupus erythematosus, Behçet's disease, tuberculosis, and HIV infection [2].

The differential diagnosis among LVA, pseudoaneurysm, and diverticulum was carefully considered using multimodality imaging. The absence of myocardial continuity on CT and the preserved myocardial signal on cardiac magnetic resonance imaging supported the diagnosis of an acquired aneurysm rather than a congenital diverticulum. A traumatic etiology was considered unlikely given the absence of a relevant trauma history and the imaging characteristics. Diverticula are generally considered congenital outpouchings of the ventricular wall composed of myocardial tissue. The width of the neck varies among cases, and contractile behavior differs depending on the histologic type. Muscular diverticula typically exhibit synchronous contraction with the ventricle and contain all myocardial layers, whereas fibrous diverticula tend to show akinetic or dyskinetic motion and consist mainly of fibrous connective tissue [3].

In contrast, our case demonstrated myocardial discontinuity and a fissure‐like morphology, supporting the diagnosis of acquired LVA rather than a diverticulum. Nevertheless, given the rarity of such lesions and the absence of prior myocardial infarction, the possibility of a diverticulum cannot be entirely excluded. Thus, the indication for surgery was reinforced by the appearance of a new pericardial effusion, suggesting a risk of rupture. Intraoperative findings of a localized, contractile outpouching with a relatively wide neck (approximately 10 mm) and no pericardial rupture further supported the diagnosis of an acquired saccular aneurysm.

Standard repair typically involves median sternotomy with left ventriculotomy, whereas alternative techniques, including left anterior mini‐thoracotomy, trans‐left‐atrial closure, and bilateral mini‐thoracotomy under CPB with cardiac arrest, have been reported [4, 5, 6]. However, previously reported thoracotomy‐based approaches were generally performed under cardiac arrest, which was avoided in the present case.

In addition, trans‐left atrial and bilateral mini‐thoracotomy techniques would have required right thoracotomy, aortic cross‐clamping, and partial dissection of the retrosternal gastric conduit, thereby increasing the risk of conduit injury.

Preoperative imaging confirmed that the aneurysm was located in a position accessible via a left mini‐thoracotomy. CPB could be safely established through femoral cannulation, and the aneurysm morphology allowed exclusion under on‐pump beating conditions. This case highlights that a left mini‐thoracotomy can serve as a safe and practical alternative when median sternotomy is contraindicated due to the presence of a retrosternal gastric conduit.

Although an off‐pump closure could have avoided CPB, we used CPB to maximize safety and ensure a secure closure. Cardiac arrest and hypothermic ventricular fibrillation arrest were considered but ultimately avoided. Manipulation of the ascending aorta posed substantial risks in the presence of a retrosternal gastric conduit, and the patient's advanced age and frailty discouraged additional invasiveness. While thoracotomy‐based repairs for LVAs have been described, performing such a procedure in a patient with a retrosternal gastric conduit is extremely uncommon. In this setting, median sternotomy carries a high risk of conduit injury, and our case illustrates that a minimally invasive left mini‐thoracotomy can serve as a safe and effective alternative surgical strategy.

Preoperative CT and TTE obtained in the operative position were invaluable for selecting the thoracotomy interspace. The left mini‐thoracotomy provided excellent direct exposure of the inferior LV wall and may also permit concomitant coronary artery bypass grafting through the same incision in selected patients. This experience highlights that MICS via a left mini‐thoracotomy can be a safe and practical alternative when median sternotomy is undesirable or impractical.

## 6 Conclusions

In patients with prior esophagectomy and retrosternal gastric conduit, conventional sternotomy may pose substantial risks. This case demonstrates that the MICS procedure via left mini‐thoracotomy can be performed safely and effectively, offering an important teaching point for tailoring surgical strategies in complex anatomical settings.

## Author Contributions

**Junya Kitaura:** conceptualization, visualization, writing – original draft. **Tomokuni Furukawa:** conceptualization, supervision, writing – review and editing. **Kazuki Maeda:** data curation, supervision.

## Funding

The authors have nothing to report.

## Ethics Statement

The authors have nothing to report.

## Consent

Written informed consent for publication was obtained from the patient.

## Conflicts of Interest

The authors declare no conflicts of interest.

## Data Availability

Data sharing not applicable to this article as no datasets were generated or analysed during the current study.

## References

[1] R. Lorusso , M. Matteucci , S. Lerakis , et al., “Postmyocardial Infarction Ventricular Aneurysm: JACC Focus Seminar 5/5,” Journal of the American College of Cardiology 83, no. 19 (2024): 1917–1935, 10.1016/j.jacc.2024.02.044. 38719371

[2] M. Paul , M. Schäfers , M. Grude , et al., “Idiopathic Left Ventricular Aneurysm and Sudden Cardiac Death in Young Adults,” Europace 8, no. 8 (2006): 607–612, 10.1093/europace/eul074. 16864613

[3] M. B. Srichai , E. M. Hecht , D. C. Kim , and J. E. Jacobs , “Ventricular Diverticula on Cardiac CT: More Common Than Previously Thought,” AJR. American Journal of Roentgenology 189, no. 1 (2007): 204–208, 10.2214/AJR.06.1223. 17579172

[4] O. Babliak , D. Babliak , V. Lazoryshynets , K. Revenko , Y. Melnyk , and O. Stohov , “Left Ventricular Aneurysm Repair Through the Left Anterior Minithoracotomy,” Innovations (Phila) 20, no. 3 (2025): 272–275, 10.1177/15569845251333424. 40369844

[5] U. Da Col , I. Di Bella , E. Ramoni , et al., “Repair of Posterior Left Ventricular Aneurysm Through Transatrial Approach,” Journal of Cardiac Surgery 25, no. 1 (2010): 23–25, 10.1111/j.1540-8191.2009.00819.x. 19549045

[6] Y. Kanazawa , S. Saito , I. Shibasaki , et al., “Minimally Invasive Cardiac Surgery via Bilateral Thoracotomy in Treatment of Left Ventricle Aneurysm: A Case Report,” Surgical Case Reports 9, no. 1 (2023): 60, 10.1186/s40792-023-01640-9. 37052756 PMC10102262

